# A new species of *Hemiphyllodactylus* Bleeker, 1860 (Squamata, Gekkonidae) from Son La Province, Vietnam

**DOI:** 10.3897/zookeys.1268.177040

**Published:** 2026-02-04

**Authors:** Hong Bich Ha, Tuoi Thi Hoang, Manh Dac Nguyen, Nghia Van Ha, Vinh Quang Luu

**Affiliations:** 1 College of Forestry Biotechnology, Vietnam National University of Forestry, Hanoi, Vietnam College of Forestry Biotechnology, Vietnam National University of Forestry Hanoi Vietnam; 2 Faculty of Forest Resources and Environmental Management, Vietnam National University of Forestry, Hanoi, Vietnam Faculty of Forest Resources and Environmental Management, Vietnam National University of Forestry Hanoi Vietnam; 3 Ha Gia Thanh Company Limited, Hanoi, Vietnam Ha Gia Thanh Company Limited Hanoi Vietnam

**Keywords:** Gecko, morphology, phylogeny, Southeast Asia

## Abstract

An integrative analysis revealed a new species of the *Hemiphyllodactylus typus* group collected from local house walls and surrounding karst formations in Pa Kha 1 Village, Long Phieng Commune, Son La Province, northwestern Vietnam. *Hemiphyllodactylus pakhaensis***sp. nov**. is recovered within Clade 4 of the *typus* group, exhibiting an uncorrected pairwise distance greater than 6.65% from all other congeners based on a 1,043 base pair segment of the ND2 gene. It can be distinguished from its congeners by body size, chin scales, circumnasal scales, dorsal and ventral scales. A multiple factor analysis using normalized morphometric, meristic, and categorical character types recovered its unique, non–overlapping placement in morphospace as statistically significantly different from closely related species in the Clade 4. The discovery and description of this new *Hemiphyllodactylus* species represent the first record of the Clade 4 from Vietnam and increase the number of species of this genus recorded in the country to 12.

## Introduction

*Hemiphyllodactylus* is a diverse gekkonid genus with at least 74 known species (Uetz et al. 2025), concentrated in karst and montane landscapes in Southeast Asia, with Vietnam emerging as a critical biodiversity hotspot (Grismer et al. 2013; Yushchenko et al. 2023). The genus has experienced rapid taxonomic expansion, with species count increasing dramatically through integrative research and more field surveys. An integrative taxonomic approach has revealed that these interesting “half leaf-fingered” geckos are far more diverse than previously recognized, currently composed of at least 19 species with 10 additional new species-level lineages identified across Southeast Asia (Grismer et al. 2013). In Vietnam alone, 11 *Hemiphyllodactylus* have been documented, with many new species discovered in recent years from regions like Hoa Binh, Son La, Ha Giang, and Tuyen Quang provinces (Nguyen et al. 2020; Do et al. 2020; Luu et al. 2023, 2024, 2025). Researchers emphasize that the current taxonomy likely underestimates the herpetological diversity, with karst ecosystems being especially rich in highly endemic and site-specific species, as demonstrated by many recent taxonomic discoveries (Luu et al. 2024; Neang et al. 2024). Continued field surveys are crucial for understanding the full biodiversity of karst ecosystems, including this gecko genus, with additional species discoveries are likely exists throughout the region (Neang et al. 2024).

Based on phylogenetic analyses presented in previous studies, there is a group comprising Clade 4 of the *Hemiphyllodactylus typus* group (Grismer et al. 2017, 2018, 2020; Agung et al. 2021, 2022; Zhou et al. 2024; Wang et al. 2025). Members of this Clade are known only from China, Myanmar, and Thailand. This group currently comprises 13 species including six species from China (*H. diaoluoshanensis* Wang, Qi, Zhang, Zheng, Li, Song, Xie, Li & Wang, 2025, *H. jianfenglingensis* Wang, Qi, Zhang, Zheng, Li, Song, Xie, Li & Wang, 2025, *H. jinpingensis* Zhou & Liu, 1981, *H. menglianensis* Zhou, Li, Yuan, Cui, Liu & Rao, 2024, *H. mengsongcunensis* Zhou, Li, Yuan, Cui, Liu & Rao, 2024, and *H. simaoensis* Agung, Chornelia, Grismer, Grismer, Quah, Lu, Tomlinson & Hughes, 2022); six species from Myanmar (*H. linnwayensis* Grismer, Wood, Thura, Zin, Quah, Murdoch, Grismer, Li, Kyaw & Lwin, 2017, *H. montawaensis* Grismer, Wood, Thura, Zin, Quah, Murdoch, Grismer, Li, Kyaw & Lwin, 2017, *H. ngwelwini* Grismer, Wood, Quah, Thura, Oaks & Lin, 2020, *H. tonywhitteni* Grismer, Wood, Thura, Zin, Quah, Murdoch, Grismer, Li, Kyaw & Lwin, 2017, *H. uga* Grismer, Wood, Zug, Thura, Grismer, Murdoch, Quah & Lin, 2018, and *H. ywanganensis* Grismer, Zug, Wood, Thura, Grismer, Murdoch, Quah & Lin, 2018); and one species from Thailand (*H. chiangmaiensis* Grismer, Wood & Cota, 2014).

**Figure 1.**
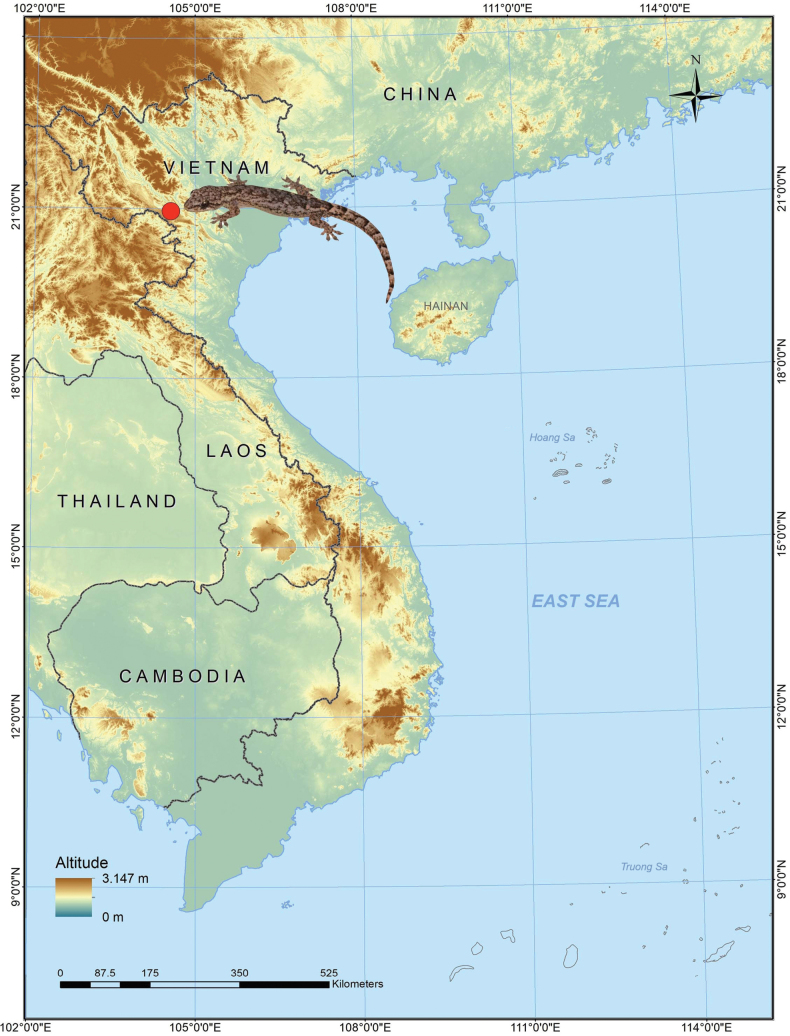
Type locality of *Hemiphyllodactylus pakhaensis* sp. nov. Pa Kha 1 Village, Long Phieng Commune, Son La Province, northwestern Vietnam (red dot).

During herpetological surveys in northwestern Vietnam in September 2023 in Pa Kha 1 Village, Chieng Tuong Commune, Yen Chau District (currently known as Long Phieng Commune), Son La Province (Fig. 1), we collected four specimens of an unknown species belonging to the half leaf-fingered gecko of the genus *Hemiphyllodactylus*. Our initial analysis showed that this new population could be assigned to Clade 4 of the *H. typus* group, but it can be distinguished from other known congeners based on morphometric, meristic, categorical, and genetic divergence. We herein describe a new species from Son La Province.

## Materials and methods

### Sampling

A survey was carried out in September 2023 in Pa Kha 1 Village, Long Phieng Commune, Son La Province, northwestern Vietnam. Four individuals were collected, photographed alive, and then anaesthetized and euthanized in a closed vessel with a piece of cotton wool saturated with ethyl acetate (Simmons 2002), fixed in 85% ethanol, and subsequently stored permanently in 70% ethanol. Liver tissues were preserved separately in 70% ethanol for genetic analysis. Specimens and tissues were deposited in the collection of the Vietnam National University of Forestry (**VNUF**), Hanoi, Vietnam.

### Morphological data

Terminology follows Zug (2010), Zhou et al. (2024), Grismer et al. (2025), and Luu et al. (2025). Measurements were taken with a digital caliper (Mitutoyo CD-6”PSX) to the nearest 0.1 mm. The following character abbreviations and descriptions were used: **SVL**: snout–vent length (from the tip of snout to the vent); **TaL**: tail length (from the vent to the tip of the tail, original and regenerated); **TrunkL**: trunk length (from the posterior margin of the forelimb at its insertion point on the body to the anterior margin of the hindlimb insertion point on the body); **HL**: head length (from the posterior margin of the lower jaw retroarticular process to the tip of the snout); **HW**: head width (measured at the angle of the jaws); **ED**: eye diameter (the greatest horizontal diameter of the eyeball); **SnEye**: snout–eye length (from anteriormost margin of the eyeball to the tip of snout); **NarEye**: nares–eye length (from the anterior margin of the eyeball to the posterior margin of the external nares); **SnW**: internarial width (measured between the nares across the rostrum).

The following meristic characters were counted using a zoom stereomicroscope (Olympus SZ60) with LED light. Bilateral scale counts were given as left/right: **CS**: chin scales (the number of scales contacting the infralabials and mental from the suture between the second and third infralabials on the left side to the suture between the second and third infralabials on the right side); **CN**: circumnasal scales (the number of scales abutting the external naris, exclusive of the rostral and first supralabial); **IS**: intersupranasals (the number of scales between the supranasals); **SL**: supralabial scales (number of enlarged scales bordering the mouth on the upper jaw from the rostral to a point in line with the posterior margin of the orbit); **IL**: infralabial scales (the number of enlarged scales bordering the mouth on the lower jaw from the mental to a point in line with the posterior margin of the orbit); **VS**: ventral scales (the number of longitudinal ventral scales at mid-body contained within one eye diameter); **DS**: dorsal scales (the number of longitudinal dorsal scales at mid–body contained within one eye diameter); lamellae formulae determined as the number of U-shaped, subdigital lamellae (split and single) on the digital pads on digits II–V of both hands and feet; **FL1**: the number of subdigital lamellae wider than long on the first finger; **TL1**: the number of subdigital lamellae wider than long on the first toe; the total number of precloacal and femoral pores (i.e. the contiguous or discontinuous rows of femoral and precloacal pore-bearing scales); **CloacS**: the number of cloacal spurs.

The categorical color pattern characters evaluated were the presence or absence of the following characters: bold dark markings/reticulate pattern on dorsal head (**BodarPatDorHead**); dorsolateral pale-colored spots on trunk (**DorLatLiSpts**); a broad dark mid–dorsal stripe (**BroDarMidDorStrip**); a dark ventrolateral stripe on trunk (**DarVenLaStrip**); bold dark markings on dorsal surfaces of limbs (**BodarMarkDorLimbs**).

### Genetic data

A total of 1,043 base pairs of the NADH dehydrogenase subunit 2 (ND2) gene from 34 sequences of 13 species within Clade 4, (Zhou et al. 2024; Wang et al. 2025) were downloaded from GenBank, and four new sequences from specimens collected from Pa Kha 1 Village (VNUF R.2025.31–34), were included in the phylogenetic analyses (Suppl. material 1). *Hemiphyllodactylus harterti* (Werner, 1900) of the *H. harterti* group was used to root the tree (follows Grismer et al. 2013). For the molecular phylogenetic analyses, genomic DNA was extracted from liver tissues stored in ethanol following the standard protocols of the DNeasy blood and tissue kit, Qiagen (Germany). A fragment of the mitochondrial gene ND2 (NADH dehydrogenase subunit 2) was amplified and sequenced using the primer pair, ND2f101A (5’-CAACAGAAGCCACAACAAAAT -3’) and HemiR (5’-GAAGAAGAGGCTTGGKAGGCT-3’) (Nguyen et al. 2013). The PCR volume consisted of 20 µl (1 µl each primer 10 µM, 7 µl deionized water, 10 µl of Taq mastermix 2× and 1 µl DNA template 100 ng/µl). PCR conditions were: 95 °C for 5 min, followed by 42 cycles: 95 °C for 30 s, 50 °C for 45 s, and 72 °C for 60 s with a final elongation step for 6 min at 72 °C. PCR products were visualized using electrophoresis through a 1.2% agarose gel, DNA marker 100 bp, 1× TAE and stained with RedSafe Nucleic Acid Staining Solution and photographed under UV light of Geldoc system (Quantum CX5, Vilber, France). Successful amplifications were purified using innuPREP Gel Extraction Kit (Analytik Jena, Germany). Cleaned PCR products were sent to 1^st^ Base (Malaysia) for sequencing in both directions. Sequences were aligned using Muscle in MEGA 12 (Kumar et al. 2024).

### Phylogenetic analyses

Maximum likelihood (ML) and Bayesian inference (BI) analyses were used to estimate the phylogenetic relationships among the sampled sequences in the alignment. An ML phylogeny was estimated using the IQ–TREE webserver (Nguyen et al. 2015; Trifinopoulos et al. 2016) preceded by the selection of substitution models using the Bayesian Information Criterion (BIC) in Model Finder (Kalyaanamoorthy et al. 2017), which supported HKY+F+G4 as the best fit model of evolution for ND2 codon position 1, TPM3+F+G4 for position 2, and TIM2+F + G4 for position 3. One thousand bootstrap pseudoreplicates via the ultrafast bootstrap (UFB; Hoang et al. 2018) approximation algorithm were employed and nodes having ML UFB values of 95 and above were considered strongly supported (Bui et al. 2013) and nodes having 90–94 were considered well-supported.

The Bayesian inference (BI) analysis was carried out using MrBayes 3.2.7 (Ronquist et al. 2012) XSEDE on the CIPRES Science Gateway (Cyberinfrastructure for Phylogenetic Research; Miller et al. 2010), employing the GTR+I+G model of evolution to all partitions using default priors. Two independent Markov chain Monte Carlo (MCMC) simulations were performed, each with four chains, three hot and one cold. The MCMC simulation was run for 10 million generations, sampled every 1,000 generations, and the first 25% of each run was discarded as burn-in. Convergence and stationarity of all parameters from both runs were checked using Tracer v. 1.7.1 (Rambaut et al. 2014) to ensure effective sample sizes (ESS) were above 200. Post-burn-in sampled trees from both runs were combined using the sumt function in MrBayes and a 50% majority rule consensus tree was constructed. Nodes with Bayesian posterior probabilities (BPP) of 0.95 and above were considered strongly supported (Huelsenbeck et al. 2001; Wilcox et al. 2002) and nodes from 0.90–0.94 were considered well-supported. MEGA12 (Kumar et al. 2024) was used to calculate uncorrected pairwise sequence divergences (p-distance) among the species using the complete pairwise deletion option (Table 1).

**Table 1.** Uncorrected pairwise sequence divergences (p-distances) among mitochondrial ND2 lineages of Clade 4 of the *Hemiphyllodactylus typus* group.

		1	2	3	4	5	6	7	8	9	10	11	12	13	14
1	*H. ngwelwini*														
2	*H. jinpingensis*	0.1937													
3	*H. simaoensis*	0.1024	0.1961												
4	*H. ywanganensis*	0.0861	0.2044	0.0944											
5	*H. linnwayensis*	0.0913	0.2016	0.0894	0.0339										
6	*H. uga*	0.0989	0.2046	0.1032	0.0434	0.0460									
7	*H. tonywhitteni*	0.0979	0.1886	0.1001	0.0905	0.0999	0.1013								
8	*H. montawaensis*	0.1048	0.1910	0.0925	0.0945	0.0986	0.1051	0.0597							
9	*H. chiangmaiensis*	0.1310	0.1763	0.1311	0.1412	0.1487	0.1401	0.1330	0.1321						
10	*H. pakhaensis* sp. nov.	0.1059	0.1992	0.0665	0.1085	0.1116	0.1178	0.1149	0.1175	0.1443					
11	*H. diaoluoshanensis*	0.1597	0.2017	0.1433	0.1686	0.1723	0.1714	0.1598	0.1529	0.1633	0.1602				
12	*H. jianfenglingensis*	0.1497	0.1933	0.1377	0.1533	0.1553	0.1576	0.1529	0.1459	0.1629	0.1512	0.0716			
13	*H. menglianensis*	0.1048	0.2012	0.0578	0.0920	0.0936	0.1116	0.1002	0.1060	0.1382	0.0733	0.1513	0.1347		
14	*H. mengsongcunensis*	0.1746	0.1972	0.1706	0.1645	0.1661	0.1673	0.1543	0.1575	0.1717	0.1773	0.1776	0.1633	0.1686	

### Statistical analyses

Based on the phylogeny of *Hemiphyllodactylus* reported by Zhou et al. (2024) and Wang et al. (2025), Clade 4 comprises lineages from China, Myanmar, and Thailand, to which the new populations belong (see below). For the statistical analyses, the newly discovered population was distinguished from its closest relatives within Clade 4, including *H. menglianensis* and *H. simaoensis*, both from China. Raw morphological data used for the analyses were obtained from the population at Pa Kha 1 (*n* = 4), *H. menglianensis* (*n* = 4), and *H. simaoensis* (*n* = 2), as well as data available from previous studies (Agung et al. 2022; Zhou et al. 2024) (Suppl. material 2).

All statistical analyses were conducted using R v. 4.4.2 (R Core Team 2024). To eliminate potential allometric effects in the morphometric characters (see Chan and Grismer 2022), size correction was applied using the following equation:

X_adj_ = log(X)–β[log(SVL)–log(SVL_mean_)]

where:

X_adj_ = adjusted value;

X = measured value;

β = unstandardized regression coefficient for each population;

SVL_mean_ = overall average SVL of all populations (Thorpe 1975, 1983; Turan 1999; Lleonart et al. 2000).

A one-way analysis of variance (ANOVA) was conducted on transformed morphometric and meristic characters with statistically similar variances (i.e., p values > 0.05 in a Levene’s test) to determine statistically significant mean differences (p < 0.05) across the data set. Characters that were significantly different were further analyzed using Tukey’s HSD post hoc test to estimate pairwise differences among populations (significance level of p < 0.05 was used). Boxplots and violin plots were generated to visualize the range, mean, and degree of variation among species with significantly different characters.

Morphospatial relationships among species were visualized using principal component analysis (PCA) based on eight size-corrected morphometric characters (SVL, TrunkL, HL, HW, ED, SnEye, NarEye, SnW) and seven meristic characters (CS, CN, IS, SL, IL, VS, DS). PCA was conducted using the ADEGENT package in R (Jombart et al. 2010) to assess whether the distribution of individuals in morphospace aligned with the putative species boundaries inferred from molecular phylogenetic analyses and supported by univariate statistics. The PCA, implemented via the “prcomp()” function in R, is an unsupervised method that captures overall variance among populations without assuming predefined groupings. Following the PCA, a Discriminant Analysis of Principal Components (DAPC) was conducted to corroborate and further discriminate morphospatial differences among the putative species.

A multiple factor analysis (MFA) was applied using the FactorMineR package (Husson et al. 2017) and visualized with Factoextra (Kassambara and Mundt 2017) to evaluate morphospace similarities and differences between individuals from the population at Pa Kha 1 and their closely related congeners. The MFA was based on a concatenated dataset comprising eight size-corrected morphometric and seven meristic dataset, and five categorical traits (BodarPatDorHead, DorLatLiSpts, BroDarMidDorStrip, DarVenLaStrip, and BodarMarkDorLimbs), creating a nearly total evidence morphological dataset (Suppl. material 2). MFA is a global, unsupervised multivariate analysis that allows the simultaneous integration of both qualitative and quantitative variables (Pagès 2015), making it particularly well–suited for studies involving diverse data types (Grismer 2025). In MFA, all data types were standardized to prevent any single type from disproportionately influencing the analysis (see Luu et al. 2024 for further methodological details). A Permutational Multivariate Analysis of Variance (PERMANOVA) was conducted to test whether the centroid positions and group clusters of each species differed significantly based on MFA load scores from dimensions 1–5 (Anderson 2017; Oksanen et al. 2020). A Euclidean dissimilarity matrix was generated, and significance was assessed using 50,000 permutations.

## Results

### Phylogenetic analyses

Phylogenetic analyses using both Maximum Likelihood (ML) and Bayesian Inference (BI) methods were conducted based on an alignment of 1,043 nucleotide positions of the mitochondrial ND2 gene. The alignment comprised 852 constant characters and 191 variable positions. Among the variable sites, 24 were parsimony-uninformative and 167 were parsimony-informative. Maximum Parsimony analysis yielded a tree with a Consistency Index (CI) of 0.658, a Retention Index (RI) of 0.858, and a Rescaled Consistency Index (RC) of 0.565. These statistics confirm a strong phylogenetic signal in the ND2 dataset, supporting the stability of the clades recovered in ML and BI analyses.

**Figure 2.**
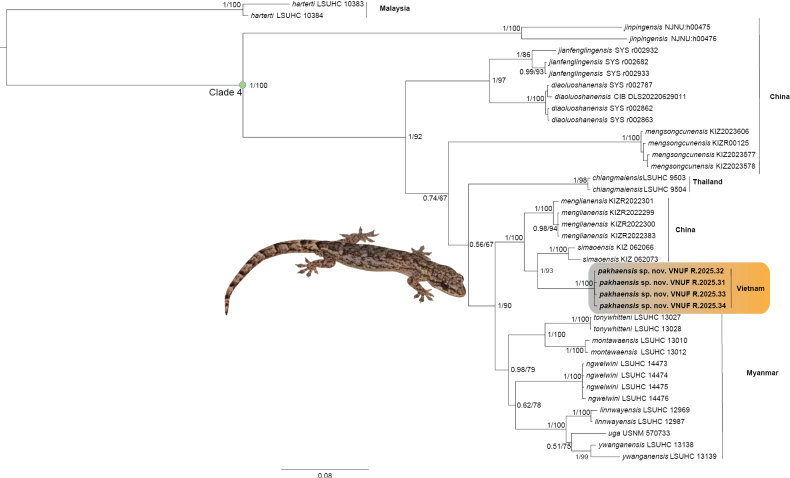
Maximum likelihood consensus tree of Clade 4 of *Hemiphyllodactylus* with Bayesian posterior probabilities (BPP) and Ultrafast Bootstrap support (UFB) value coding at the nodes, respectively.

The congruent topologies derived from both analytical approaches robustly place the *Hemiphyllodactylus* population from Pa Kha 1 within Clade 4, where it is recovered as the sister *H. simaoensis* with high statistical support values from both analyses (PP = 1, UFB = 93) (Fig. 2). Intrapopulation genetic variation within the four Pa Kha 1 specimens showed no uncorrected p–distance observed. Conversely, the interspecific divergence between the Pa Kha 1 population and all other known congeners was marked by a minimum uncorrected p-distance of 6.65%. This level of genetic separation surpasses the divergence values documented between several established, distinct species pairs within this genus, for example: 5.78% between *H. menglianensis* and *H. simaoensis*, 4.34% between *H. ywanganensis* and *H. uga*, 3.39% between *H. ywanganensis* and *H. linnwayensis*, 4.60% between *H. linnwayensis* and *H. uga*, and 5.97% between *H. montawaensis* and *H. tonywhitteni* (Table 1). Thus, the relatively large genetic differentiation strongly suggests that the Pa Kha 1 population represents a cryptic species, likely an undescribed taxon, distinct from all currently recognized *Hemiphyllodactylus* species. Furthermore, this population exhibits a unique suite of morphological characteristics not encountered in any described congeners (as elaborated further below).

### Statistical analyses

**Figure 3.**
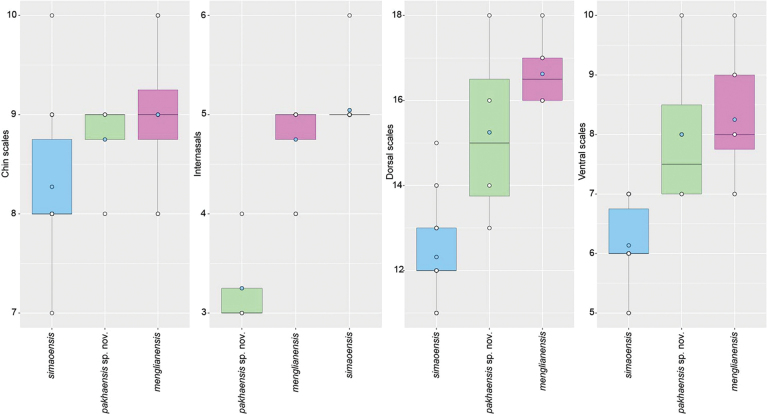
Boxplot comparisons of significantly different meristic characters between *Hemiphyllodactylus pakhaensis* sp. nov. and closely related *Hemiphyllodactylus* species (*H. simaoensis* and *H. menglianensis*) of Clade 4. Blue circles are means and the black horizontal bars are medians.

**Figure 4.**
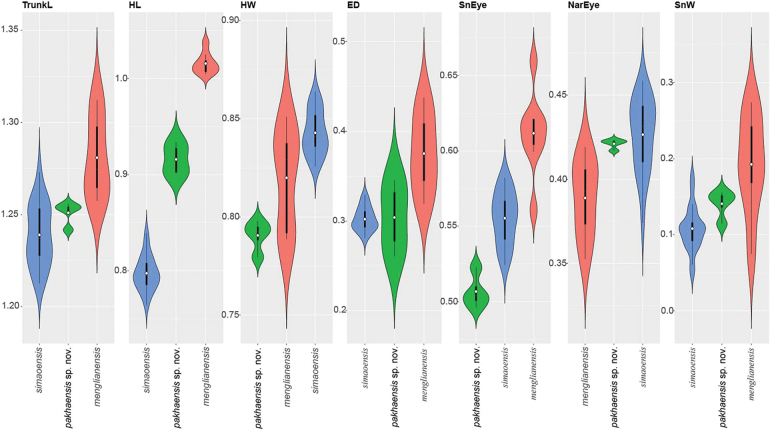
Violin plots of the significantly different morphometric characters between *Hemiphyllodactylus pakhaensis* sp. nov. and closely related *Hemiphyllodactylus* species (*H. simaoensis* and *H. menglianensis*) overlain with box plots showing the range, frequency, mean (white dot), and 50% quartile (black rectangle) of the size-adjusted morphometric and meristic characters.

The ANOVA and TukeyHSD post hoc and Welch’s F-test and Games-Howell post hoc tests of the adjusted morphometric and meristic characters were consistent with the phylogenetic results and the high degree of pairwise genetic distance among the species in recovering a number of statistically significant mean differences between the Pa Kha 1 population and closely related species. Variation in all morphometric and metric characters are visualized in Figs 3, 4 (Suppl. material 3).

**Figure 5.**
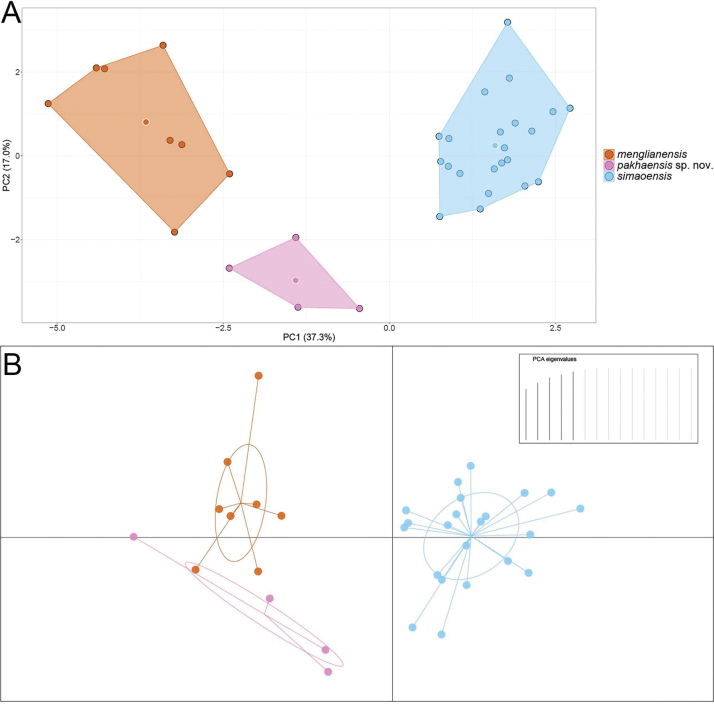
**A**. Principal component analysis (PCA) of *Hemiphyllodactylus pakhaensis* sp. nov. and closely related *Hemiphyllodactylus* species (*H. simaoensis* and *H. menglianensis*) showing their morphospatial relationships along the first two principal components; **B**. Discriminant analysis of principal components (DAPC) based on retention of the first five PCs accounting for 79.2% of the variation.

The clustering of species in the PCA of closely related species (*H. simaoensis* and *H. menglianensis*) of Clade 4 showed the first two principal components (PC1 and PC2) recovered 54.3% of the variation in the normalized morphometric and meristic data set (Fig. 5A). PC1 accounted for 37.3% of the variation in the data set and loaded most heavily for head length (HL), dorsal scales (DS), ventral scales (VS), eye diameter (ED), trunk length (TrunkL), and internarial width (SnW). PC2 accounted for an additional 17.0% of the data set and loaded most heavily for: snout-eye length (SnEye), infralabial scales (IL), supralabial scales (SL), head width (HW), and circumnasal scales (CN) (Fig. 5A, Suppl. material 4). In the PCA, the new *Hemiphyllodactylus* population from Pa Kha is widely separated from the closely related species *H. menglianensis* and *H. simaoensis*. It is uniquely isolated by its low PC2 scores, demonstrating a distinctive combination of head proportions and scale counts and an overall size and general meristics data profile that falls squarely between the larger *H. menglianensis* and the smaller *H. simaoensis*. The new population is well-separated from two closely related species in the DAPC (Fig. 5B).

**Figure 6.**
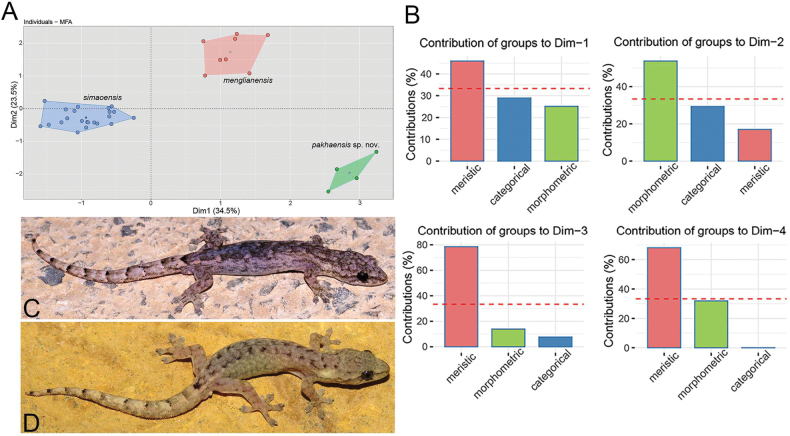
**A**. MFA scatter plot showing the morphospatial relationships *Hemiphyllodactylus pakhaensis* sp. nov. and closely related *Hemiphyllodactylus* species (*H. simaoensis* and *H. menglianensis*); **B**. Bar graphs showing the percent contribution of each data type to the MFA for the first four dimensions that account for 76.5% of the variation in the data set. The dashed red line in the bar graphs indicates the expected average value if the contributions of each data type were equal; **C**. The male holotype, VNUF R. 2025.32 from Son La, Vietnam; **D**. The male holotype of *H. simaoensis*, KIZ 062064, from Yunan, China (photo: Ade Prasetyo Agung).

The MFA analysis revealed that the new Pa Kha 1 population is clearly separated from the two closely related species within Clade 4 along Dim-1, which accounted for 34.5% of the total variation, and Dim-2, which explained an additional 23.5% of the variation in the dataset (Fig. 6A, Suppl. material 4). Meristic characters contributed most of the variation along Dims-1, 3, 4 and morphometric data contributed to the majority of the variation along Dim-2. (Fig. 6B, Suppl. material 4). In addition, the PERMANOVA analysis demonstrated that the Pa Kha 1 population differs significantly in morphospace from *H. menglianensis* and *H. simaoensis* (Table 2).

**Table 2.** Summary statistics from the PERMANOVA analysis from the loadings of the MFA comparing *Hemiphyllodactylus pakhaensis* sp. nov. to closely related *Hemiphyllodactylus* species (*H. simaoensis* and *H. menglianensis*). Significant differences in bold. Sig: Significance levels. *: p < 0.05; **: p < 0.01; ***: p < 0.001 (50 000 permutations).

Species pairs	F.Model	R2	p.value	p.adjusted	significance
*H. pakhaensis* sp. nov. – *H. menglianensis*	20.6049	0.673255	**0.00186**	**0.00558**	*
*H. pakhaensis* sp. nov. – *H. simaoensis*	44.86525	0.651493	**0.0001**	**0.0003**	**
*H. menglianensis* – *H. simaoensis*	87.71051	0.758017	**0.00002**	**0.00006**	***

### Taxonomy

#### Hemiphyllodactylus pakhaensis sp. nov.

Taxon classificationAnimaliaSquamataGekkonidae

566AFFA2-481A-5D90-8CFF-EDB227F7E20B

https://zoobank.org/A5A41376-E445-4FA3-A555-841BEE354DE8

**Figure 7.**
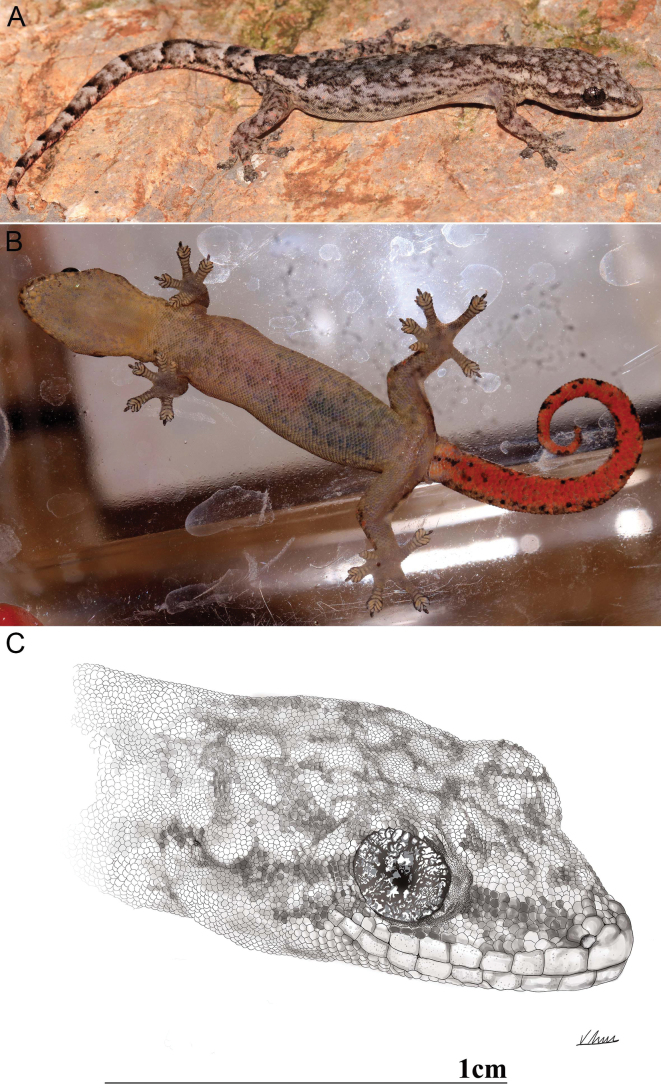
Dorsal (**A**) and ventral (**B**) views of adult male holotype VNUF R.2025.32 (Field No. Sonla02) *Hemiphyllodactylus pakhaensis* sp. nov. in life; **C**. Drawing of the holotype’s head in dorsolateral view.

**Figure 8.**
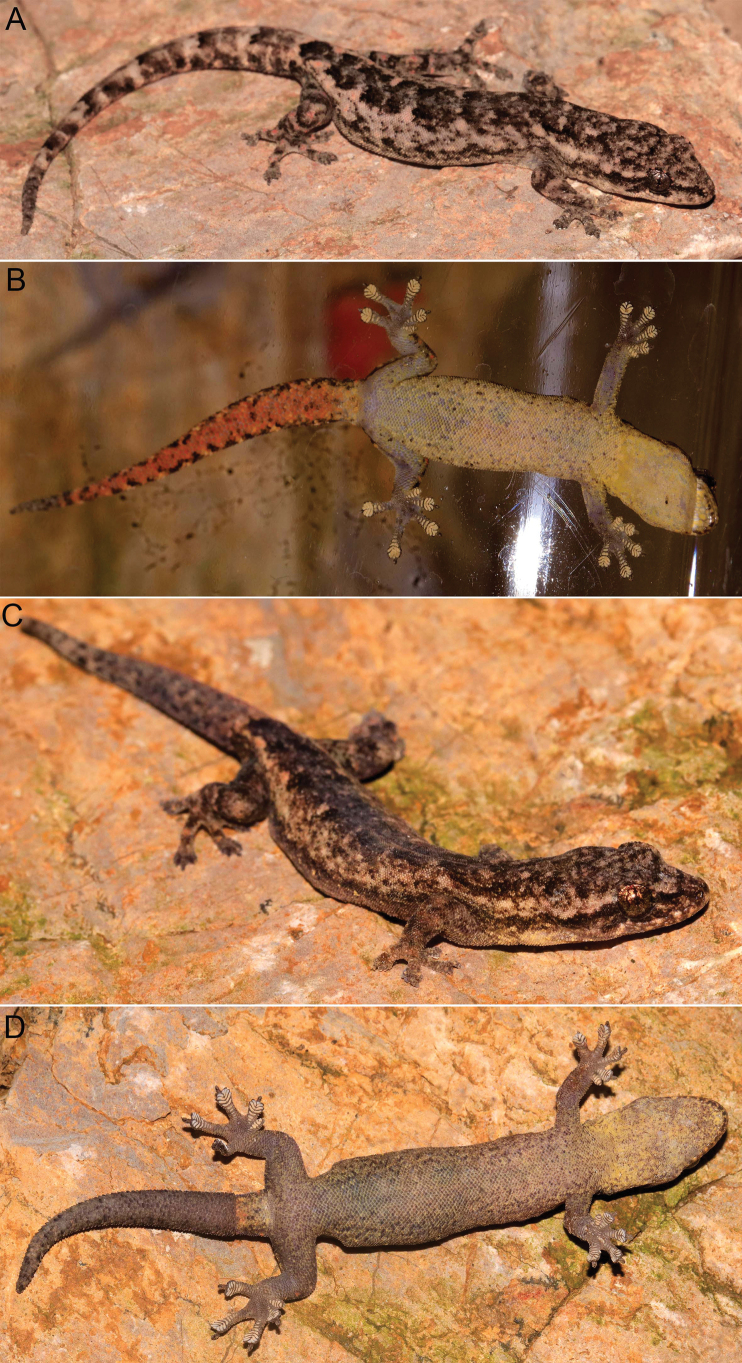
Paratypes of *Hemiphyllodactylus pakhaensis* sp. nov. in life: dorsal (**A**) and ventral (**B**) views of the paratype VNUF R.2025.31; dorsal (**C**) and ventral (**D**) views of the paratype VNUF R.2025.33.

Figs 7, 8 Pakha Slender Gecko

##### Type material.

***Holotype***. • VNUF R.2025.32 (Field No. Sonla02), an adult male, collected by Vinh Quang Luu, Vilay Phimpasone and Le Duc Phan on 05 September 2023 from Pa Kha 1 Village, Long Phieng Commune, Son La Province, northwestern Vietnam (20°52'22.6"N, 104°24'03.6"E, at an elevation of 1,068 m a.s.l.). ***Paratypes***. • VNUF R.2025.31 (Field No. Sonla01), VNUF R.2025.33 (Field No. Sonla03), all adult females and VNUF R.2025.34 (Field No. Sonla04), a subadult male, collected the same day and at the same locality as holotype.

##### Diagnosis.

*Hemiphyllodactylus pakhaensis* sp. nov. differs from its congeners by the combination of the following characters: a maximum SVL of 38.28 mm; trunk not particularly elongate (TrunkL/SVL ratio 0.50–0.52); eight or nine chin scales, distinct enlarged postmentals; three or four circumnasal scales; nine or 10 supralabial scales; eight or nine infralabial scales; 7–10 ventral scales; 13–18 dorsal scales; 3(4)–4–4–4 digital lamellae formula of forefoot (II–V) and 4–4(5)–4–4 of hindfoot (II–V); 17 or 18 precloacal pores and continuous series in males, 0–19 pitted precloacal scales in females; cloacal spur single in both sexes; enlarged subcaudal scales absent; dorsal surface of head grey, with bold dark markings; a distinct dark postnasal stripe extending to at least the base of the neck, uneven dark streaks running along the flanks and ending at the base of tail; a black streak on the anterior corner, extending below the eye to the lower jaw corner; a broad dark mid–dorsal; dorsolateral pale-colored spots on trunk becoming brown posteriorly; bold dark markings on dorsal surfaces of limbs; hind limbs with brown spots; a cream-colored V-shaped postsacral mark with anteriorly projecting arms; and unpigmented caecum and gonads.

##### Description of holotype.

Adult male, SVL 35.65 mm; head triangular in dorsal profile, moderate size (HL/SVL 0.23), longer than wide (HW/HL 0.75), depressed, distinct from neck; snout moderate (SnEye/HL 0.42), round in dorsal profile; lores concave; prefrontal region flat; rostral regular rectangular, wider than mental, contacted posteriorly by two large supranasals and three intersupranasals; external nares oval, bordered rostral anteriorly, first supralabial ventrally, supranasal dorsally and two nasals posteriorly; eye large (ED/HL 0.24), pupils vertical; ear opening oblique; 9/9 supralabial scales, 8/9 infralabial scales (left/right), gradually decreasing in size towards the angle of the jaw; scales on head small, round, larger on rostrum and lores; mental triangular, in contact with first infralabials and posteriorly by two enlarged postmentals, nine chin scales touching the internal edge of infralabials and mental from the suture between the second and third infralabials on both sides, anterior pair enlarged; gular scales small, granular (Fig. 7A, B, C).

Body slender, elongated (TrunkL/SVL 0.52), compressed; dorsal scales smooth, round or oval, small, granular in 16 rows at midbody on the dorsum within one eye diameter; ventral scales smooth and flat, distinctly larger than dorsal scales, subimbricate and largest in mid-belly, seven scales within one eye diameter; 17 pore bearing femoral and precloacal scales, slightly enlarged, in a continuous row.

Fore and hind limbs relatively short, dorsum covered with granular, subimbricate scales, slightly smaller smooth scales ventrally; all digits well developed (except digit I), clawed and robustly dilated distally; digit I vestigial, clawless with transversely expanded lamellae, five on both first fingers and first toes; claws on digits II–V well developed, unsheathed; subdigital lamellae of digits II–V divided, angular and U-shaped; lamellae proximal to these transversely expanded, undivided; distal subdigital lamellae formula 4(3)–4–4–4 (fingers II–V) and 4–4–4–4 (toes II–V); relative length fingers and toes I<II<III≈V<IV.

Tail slightly shorter than SVL, swollen at base, oval in cross–section; caudal scales forming distinctly segmented; dorsal caudal scales larger than dorsal body scales, flat, subcycloid, and subimbricate; subcaudals slightly larger than dorsal caudals, not plate-like; a single enlarged cloacal spur present on each side.

##### Coloration in life.

Dorsal surface of head grey, with bold dark reticulated markings; a distinct dark postnasal stripe extending to at least the base of the neck, uneven dark streaks running along the flanks and ending at the base of tail; a black streak on the anterior corner, extending below the eye to the lower jaw corner; a broad dark mid-dorsal; dorsolateral pale-colored spots on trunk becoming brown posteriorly; bold dark markings on dorsal surfaces of limbs; hind limbs with brown spots; a cream-colored, V-shaped postsacral mark with anteriorly projecting arms; ventral surface of head cream, faint black spots on each scale; belly and underside of limbs grayish brown with larger pale black spots; dorsal surface of tail with ~10 alternating black and white bands; underside of tail red, black dots along both lateral margins; and unpigmented caecum and gonads.

##### Coloration in alcohol.

In preservative, dorsal ground color of head, body, and limbs become paler; color pattern on dorsal surface blurred; ventral surface grayish white; subcaudal faded to grayish cream.

##### Sexual dimorphism and variation.

Females differ from males by the absence of hemipenial swellings at the tail base and by having 0–19 pitted precloacal scales (vs distinct pore-bearing scales in males). The dorsal and ventral surfaces of the regenerated portion of the tail (VNUF R.2025.33) are uniformly dark grey (Fig. 8A, B). Other morphological characters of the paratypes are provided in Table 3.

**Table 3.** Mensural (in mm), meristic, color pattern, and proportions of the type series of *Hemiphyllodactylus pakhaensis* sp. nov. (/) = data unavailable. (*) = regenerated tail. For abbreviations see Materials and methods.

Character	Holotype	Paratype		
	VNUF R.2025.32 (Field No. Sonla02)	VNUF R.2025.31 (Field No. Sonla01)	VNUF R.2025.33 (Field No. Sonla03)	VNUF R.2025.34 (Field No. Sonla04)
Sex	male	female	female	male
SVL	35.65	35.08	38.28	30.47
TaL	33.98	33.94*	24.3*	24.06*
TrunkL	18.44	17.55	19.75	15.58
HL	8.16	8.05	9.45	7.33
HW	6.16	6.22	6.93	5.39
ED	1.95	1.83	2.38	1.92
SnEye	3.39	3.15	3.42	2.88
NarEye	2.67	2.62	2.78	2.47
SnW	1.3	1.43	1.41	1.4
TrunkL/SVL	0.52	0.50	0.52	0.51
HL/SVL	0.23	0.23	0.25	0.24
HW/SVL	0.17	0.18	0.18	0.18
HW/HL	0.75	0.77	0.73	0.74
SnEye/HL	0.42	0.39	0.36	0.39
NarEye/HL	0.33	0.33	0.29	0.34
ED/HL	0.24	0.23	0.25	0.26
SnW/HL	0.16	0.18	0.15	0.19
ED/NarEye	0.73	0.70	0.86	0.78
SnW/HW	0.21	0.23	0.20	0.26
CS	9	9	8	9
CN	3	3	3	4
IS	3	2	3	3
SL (left/right)	9/9	10/9	9/10	9/9
IL (left/right)	8/9	8/8	9/9	9/9
VS	7	7	8	10
DS	16	13	14	18
Lamellar formulae hands II–V (left)	4–4–4–4	4–4–4–4	4–4–4–4	4–4–4–4
Lamellar formulae hands II–V (right)	3–4–4–4	4–4–4–4	4–4–4–4	4–4–4–4
Lamellar formulae foot II–V (left)	4–4–4–4	4–4–4–4	4–5–4–4	4–5–4–4
Lamellar formulae foot II–V (right)	4–4–4–4	4–4–4–4	4–5–4–4	4–5–4–4
FL1	5	4	4	4
TL1	5	5	5	5
Total femoroprecloacal pores	17	0	19 (pitted)	18
Precloacal and femoral pore series separate (1) or continuous (0)	0	/	0	0
CloacS on each side	1/1	1/1	1/1	1/1
Subcaudals enlarged, plate–like	No	No	No	No
Dark postorbital stripe	Yes	Yes	Yes	Yes
Bold dark markings/reticulate pattern on dorsal head	Yes	Yes	Yes	Yes
Dorsolateral pale-colored spots on trunk	Yes	Yes	Yes	Yes
Broad dark mid–dorsal stripe	Yes	Yes	Yes	Yes
Dark ventrolateral stripe on trunk	Yes	Yes	Yes	Yes
Bold dark markings on dorsal surfaces of limbs	Yes	Yes	Yes	Yes
Postsacral marking anteriorly projecting arms	Yes	Yes	Yes	Yes
Caecum pigmented	No	No	No	No

##### Distribution.

*Hemiphyllodactylus pakhaensis* sp. nov. is currently known only from the type locality in Pa Kha 1 Village, Long Phieng Commune, Son La Province, northwestern Vietnam (Fig. 1).

##### Etymology.

The specific epithet *pakhaensis* is a toponym referring to the type locality of the species. We suggest the following common names: Pakha Slender Gecko (English) and Thạch sùng dẹp pa kha (Vietnamese).

##### Natural history.

**Figure 9.**
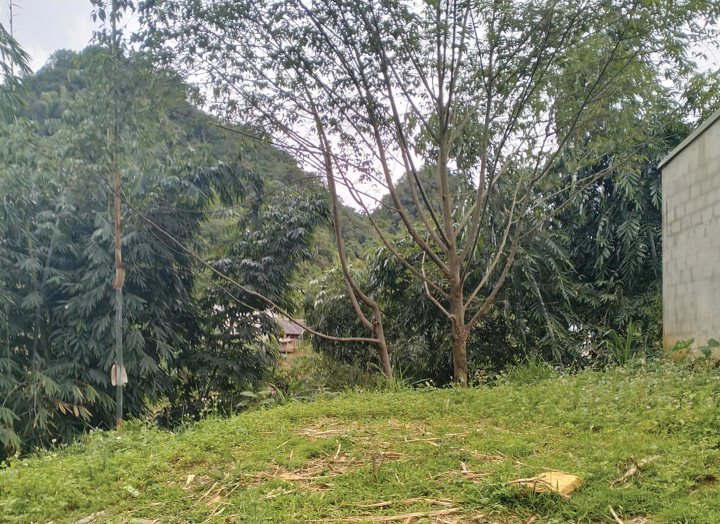
Habitat of *Hemiphyllodactylus pakhaensis* sp. nov. Pa Kha 1 Village, Long Phieng Commune, Son La Province, northwestern Vietnam.

The type series was collected on the walls of local houses between 18:30 and 22:30, at heights of ~1–2.5 m above the ground, at an elevation of 1,068 m. The surrounding habitat consisted of disturbed karst forest with small hardwood and shrub trees, situated near residential areas and road systems (Fig. 9). At the time of collection, relative humidity was 75% and air temperature was 19 °C.

##### Comparisons.

Molecular analyses indicated that *Hemiphyllodactylus pakhaensis* sp. nov. from Vietnam is nested within Clade 4, which previously comprised species known only from China, Myanmar, and Thailand. Accordingly, the new species is compared with the other 13 members of Clade 4 (Table 4) using data from recent taxonomic publications (Agung et al. 2022; Zhou et al. 2024; Wang et al. 2025).

**Table 4.** Comparisons of the new species with its 13 congeners from other members of Clade 4 (measurements in mm, * = regenerated or broken tail, other abbreviations defined in the text).

Character	*H. pakhaensis* sp. nov.	*H. chiangmaiensis*	*H. diaoluoshanensis*	*H. jianfenglingensis*	*H. jinpingensis*	*H. linnwayensis*	*H. menglianensis*	*H. mengsongcunensis*	*H. montawaensis*	*H. ngwelwini*	*H. simaoensis*	*H. tonywhitteni*	*H. uga*	*H. ywanganensis*
Maximum SVL	38.28	41.2	31	33.8	40.5	41.5	41.5	45.6	40.1	40.2	40.9	38.8	39	38
CS	8 or 9	8–12	8	9 or 10	7–10	4–6	8–10	6–10	4–7	9–13	7–10	5–8	8–10	10
Post–mentals distinctly enlarged	Yes	Yes	Yes	Yes	Yes	Yes	Yes	Yes	Yes	Yes	Yes	Yes	Yes	Yes
CN	3 or 4	3 or 4	5	5	5 or 6	5	4 or 5	5	4–6	S	5 or 6	3–5	5	5
IS	2 or 3	1–3	3	2 or 3	1–5	2	2 or 3	2 or 3	1–4	1–3	1–4	2–4	2 or 3	2 or 3
SL	9 or 10	9–11	9 or 10	9 or 10	8–12	9 or 10	9–11	8–11	8–10	8–11	8–12	8 or 9	9 or 10	9 or 10
IL	8 or 9	9–12	9 or 10	9 or 10	9–12	8	8–10	8–11	8 or 9	8–10	8–11	8	8–10	10
DS	13–18	11–21	11–13	12–14	10–15	13 or 14	16–18	11–15	13 or 14	11–14	11–15	13–16	13–15	13–15
VS	7–10	6–10	7 or 8	6	5–9	8	7–10	6–8	7 or 8	7 or 8	5–7	7–9	6–8	7 or 8
FLI	4 or 5	3 or 4	4	4	4 or 5	3 or 4	4 or 5	4 or 5	3 or 4	4	3–5	3	2 or 3	3
TLI	5	3 or 4	4	4	3–6	4 or 5	5	5	3 or 4	4	3–5	3 or 4	2 or 3	2 or 3
Lamellar formula on hand	(3 or 4)–4–4–4	3–3–(3 or 4)–3	3–3–3–3	3–3–3–3	(3 or 4)–(4 or 5)–(4 or 5)–(3 or 4)	4–4–4–4	4–(4 or 5) –(4–6)–4	(3 or 4)–(4–6) (4–7)–(4 or 5)	3–(3 or 4)–(3 or 4)–3	3–(3 or 4)–(3 or 4)–(3 or 4)	(3 or 4)–(3–5)–(3–5)–(3 or 4)	4–(4 or 5)–(4 or 5)–(4 or 5)	(2 or 3) (2 or 3)–3–3	3–3–(3 or 4)–3
Lamellar formula on foot	4–(4 or 5)–4–4	3–(3 or 4)–3–3	3–4–4–4	3–4–4–4	(3 or 4)–(4 or 5)–(4 or 5)–(4 or 5)	4–5–(4 or 5)–4	(4 or 5)–5–5–(4 or 5)	(4 or 5)–(4–6)–(4–6)–(–4 or 5)	(3 or 4)–(4 or 5)–(4 or 5) or 5)–4	(3 or 4)–(3 or 4)– (3 or 4) –(3 or 4)	(3 or 4)–(3–5)–(3–5)–(3 or 4)	(3 or 4)–(4 or 5)–(4 or 5)–(4 or 5)	3–3–3–3	3–3–3–3
Precloacal and femoral pore series separate or continuous	Continuous	Continuous	Continuous	Continuous	Separate or continuous	Continuous	Continuous	Continuous	Continuous	Separate or continuous	Continuous	Continuous	Continuous	Continuous
Total femoropre-cloacal pores in males	17 or 18	17–25	15	21	20–24	/	16–20	26–30	19–21	15–22	16–27	20–26	18–22	26
Total femoropre-cloacal pores in females	0–19 (pitted)	0	0	0	/	0	0	0	0	0	0	0–20	0	0
Cloacal spurs	1	1	1	1	1 or 2	1	1	1 or 2	1	1	1	1	1	1
Subcaudals enlarged, plate–like	No	No	No	No	No	No	No	No	No	No	No	No	No	No
Dark postorbital stripe	Yes	Yes	Yes	Yes	Yes	Yes	Yes	Yes	Yes	Yes	Yes	Variable	Yes	Yes
Dorso–lateral pale-colored spots on trunk	Yes	Yes	No	No	No	Yes	No	No	No	No	Yes, variable	No	No	Yes
Dark dorsolateral stripe on trunk	Yes	No	No	No	No	No	No	No	No	No	No, if present indistinct	No	No	No
Dark dorsal transverse blotches	No	Yes	Yes	Yes	Yes	No	Yes	Yes	No	No	Yes	No	No	No
Dorsal pattern unicolorous	No	No	No	No	No	No	/	No	No	/	No	No	No	No
Postsacral marking bearing pale-colored anteriorly projecting arms	Yes	Yes	No	No	Yes	No	Yes	Yes	Variable	Variable	Yes	Yes	No	Yes
Caecum pigmented	No	Yes	No	No	No	/	/	No	No	/	No	No	/	No
Gonads pigmented	No	Yes	No	No	No	/	/	No	No	/	No	No	/	No
TrunkL (mm)	15.58–19.75	15.2–23.1	11.6–17.9	16.4–18.3	12.5–20.4	17.7–20.4	16.8–21.0	16.5–25.2	15.6–20.9	12.7–21.4	13.9–21.0	13.7–19.2	16.2–19.4	16.9–19.1
HL (mm)	7.33–9.45	9.1–13.9	6.0–7.8	6.9–8.1	5.1–7.3	9.3–9.6	9.3–11.2	9.1–12.1	7.8–9.5	6.4–8.8	5.1–7.3	7.9–9.3	8.4–9.5	9.2–9.3
SnEye (mm)	2.88–3.42	3.1–4.7	2.4–3.3	3.0–3.3	2.8–4.0	3.7–4.0	3.5–4.6	3.4–5.0	3.4–4.1	2.8–4.5	2.9–4.2	3.2–4.6	3.6–3.8	3.8–3.9
HW (mm)	5.39–6.93	5.5–7.7	4.0–5.9	5.1–6.4	5.3–7.6	6.3–7.1	5.8–7.3	6.0–8.9	5.3–6.5	5.3–8.2	5.8–7.9	5.2–6.8	5.8–6.4	6.2–6.3
NarEye (mm)	2.47–2.78	2.2–3.3	1.9–2.1	2.1–2.6	2.2–3.0	2.8–3.0	1.6–3.1	2.4–3.7	2.3–3.1	1.9–3.2	2.3–3.2	2.3–3.1	2.5–2.9	2.7–2.9
ED (mm)	1.83–2.38	1.7–2.3	1.2–1.8	1.6–1.8	1.7–2.6	2.0–2.4	2.0–2.7	1.8–2.7	1.7–2.1	1.5–2.3	1.6–2.3	1.7–2.1	2.2–2.4	2.2
SnW (mm)	1.30–1.43	1.1–1.4	0.9–1.4	1.1–1.6	1.1–1.6	1.3	1.2–2.1	1.3–1.6	1.1–1.2	0.9–1.5	1.1–1.6	1.0–1.3	1.1–1.4	1.3–1.4
TrunkL/SVL	0.50–0.52	0.46–0.56	0.50–0.59	0.49–0.56	0.45–0.53	0.48–0.49	0.48–0.55	0.47–0.55	0.48–0.52	0.45–0.54	0.46–0.53	0.43–0.52	0.44–0.52	0.49–0.50
HL/SVL	0.23–0.25	0.25–0.43	0.23–0.26	0.23–0.24	0.17–0.19	0.23–0.25	0.26–0.28	0.25–0.29	0.23–0.26	0.19–0.26	0.16–0.20	0.24–0.27	0.23–0.24	0.24–0.26
HW/SVL	0.17–0.18	0.18–0.23	0.15–0.19	0.17–0.19	0.17–0.21	0.17–0.25	0.16–0.19	0.17–0.21	0.16–0.17	0.18–0.21	0.19–0.21	0.17–0.19	0.15–0.18	0.16–0.18
HW/HL	0.73–0.77	0.41–0.80	0.65–0.76	0.74–0.79	0.99–1.15	/	0.59–0.68	0.63–0.77	0.68–0.73	0.81–0.94	0.98–1.18	0.62–0.74	/	/
SnEye/HL	0.36–0.42	0.23–0.49	0.40–0.44	0.38–0.43	0.51–0.61	/	0.36–0.42	0.37–0.47	0.41–0.44	0.41–0.54	0.52–0.63	0.41–0.50	0.41–0.43	0.41–0.42
NarEye/HL	0.29–0.34	0.17–0.33	0.27–0.32	0.28–0.32	0.39–0.45	0.30–0.31	0.21–0.28	0.26–0.32	0.29–0.34	0.28–0.41	0.38–0.46	0.29–0.34	0.30–0.33	0.29–0.32
ED/HL	0.23–0.26	0.13–0.24	0.20–0.26	0.22–0.23	0.29–0.38	0.22–0.26	0.20–0.25	0.19–0.24	0.22–0.24	0.23–0.31	0.30–0.35	0.21–0.23	0.23–0.26	0.24
SnW/HL	0.15–0.19	0.08–0.23	0.15–0.18	0.16–0.20	0.18–0.24	0.13–0.15	0.12–0.19	0.12–0.15	0.13–0.15	0.14–0.18	0.18–0.24	0.13–0.14	0.13–0.16	0.11–0.15
ED/NarEye	0.70–0.86	0.68–0.81	0.63–0.86	0.69–0.78	0.68–0.87	1	0.71–1.12	0.64–0.77	0.68–0.76	0.63–0.84	0.70–0.86	0.65–0.75	/	/
SnW/HW	0.20–0.26	0.17–0.32	0.23–0.24	0.22–0.25	0.16–0.23	/	0.19–0.28	0.17–0.21	0.18–0.21	0.16–0.21	0.16–0.21	0.18–0.23	/	/

Morphologically, the new species differs from *H. chiangmaiensis* Grismer, Wood & Cota by having fewer infralabials (IL 8 or 9 vs 9–12), the presence of dark dorsolateral stripe on trunk (vs absent), unpigmented caecum and gonads (vs pigmented); a shorter head (HL/SVL 0.23–0.25 vs 0.25–0.43), and a narrower head (HW/SVL 0.17–0.18 vs 0.18–0.23);

from *H. diaoluoshanensis* Wang, Qi, Zhang, Zheng, Li, Song, Xie, Li & Wang by having a larger maximal size (SVL 38.28 vs 31 mm), more lamellar formula on hand ((3 or 4)–4–4–4 vs 3–3–3–3), more femoropre-cloacal pores in males (17 or 18 vs 15), and the presence of dorsolateral pale-colored spots and dark dorsolateral stripe on trunk (vs absent);

from *H. jianfenglingensis* Wang, Qi, Zhang, Zheng, Li, Song, Xie, Li & Wang by having more ventral scales (7–10 vs 6), more lamellar formula on hand ((3 or 4)–4–4–4 vs 3–3–3–3), fewer femoropre–cloacal pores in males (17 or18 vs 21), and the presence of dorsolateral pale-colored spots and dark dorsolateral stripe on trunk (vs absent); larger eyes (ED/HL 0.23–0.26 vs 0.22–0.23);

from *H. jinpingensis* Zhou & Liu by having fewer circumnasal scales (CN 3 or 4 vs 5 or 6), fewer infralabials (IL 8 or 9 vs 9–12), fewer femoropre-cloacal pores in males (17 or18 vs 20–24); a longer head (HL/SVL 0.23–0.25 vs 0.17–0.19), a narrower head (HW/HL 0.73–0.77 vs 0.99–1.15), a shorter snout–eye length (SnEye/HL 0.36–0.42 vs 0.51–0.61), and a smaller eyes (ED/HL 0.23–0.26 vs 0.29–0.38);

from *H. linnwayensis* Grismer, Wood, Thura, Zin, Quah, Murdoch, Grismer, Li, Kyaw & Lwin by having more chin scales (CS 8 or 9 vs 4–6), fewer circumnasal scales (CN 3 or 4 vs 5), the presence of femoropre-cloacal pores in males (vs absent), the presence of dark dorsolateral stripe on trunk and postsacral marking bearing pale-colored anteriorly projecting arms (vs absent), and a wider internarial width (SnW/HL 0.15–0.19 vs 0.13–0.15);

from *H. menglianensis* Zhou, Li, Yuan, Cui, Liu & Rao in terms of body ratio by having a shorter head (HL/SVL 0.23–0.25 vs 0.26–0.28), a wider head (HW/HL 0.73–0.77 vs 0.59–0.68), a longer nares–eye length (NarEye/HL 0.29–0.34 vs 0.21–0.28), and by differently statistical analyses as mentioned in Figs 3, 4, 5, 6 and Table 4;

from *H. mengsongcunensis* Zhou, Li, Yuan, Cui, Liu & Rao by having a smaller maximal size (SVL 38.28 vs 45.6 mm), fewer circumnasal scales (CN 3 or 4 vs 5), fewer femoropre-cloacal pores in males (17 or 18 vs 26–30), the presence of dorsolateral pale-colored spots and dark dorsolateral stripe on trunk (vs absent), a shorter head (HL/SVL 0.23–0.25 vs 0.25–0.29), and a wider internarial width (SnW/HL 0.15–0.19 vs 0.12–0.15);

from *H. montawaensis* Grismer, Wood, Thura, Zin, Quah, Murdoch, Grismer, Li, Kyaw & Lwin by having more chin scales (CS 8 or 9 vs 4–7), fewer circumnasal scales (CN 3 or 4 vs 4–6), fewer femoropre-cloacal pores in males (17 or 18 vs 19–21), the presence of dorsolateral pale-colored spots and dark dorsolateral stripe on trunk (vs absent), and a wider head (HW/HL 0.73–0.77 vs 0.68–0.73);

from *H. ngwelwini* Grismer, Wood, Quah, Thura, Oaks & Lin by having fewer chin scales (CS 8 or 9 vs 9–13), the presence of dorsolateral pale-colored spots and dark dorsolateral stripe on trunk (vs absent), and a narrower head (HW/HL 0.73–0.77 vs 0.81–0.94);

from *H. simaoensis* Agung, Chornelia, Grismer, Grismer, Quah, Lu, Tomlinson & Hughes in terms of body ratio by having a longer head (HL/SVL 0.23–0.25 vs 0.16–0.20), a narrower head (HW/HL 0.73–0.77 vs 0.98–1.18), a shorter snout–eye length (SnEye/HL 0.36–0.42 vs 0.52–0.63), a smaller eyes (ED/HL 0.23–0.26 vs 0.30–0.35), and by differently statistical analyses as mentioned in Figs 3, 4, 5, 6 and Table 4;

from *H. tonywhitteni* Grismer, Wood, Thura, Zin, Quah, Murdoch, Grismer, Li, Kyaw & Lwin by having more chin scales (CS 8 or 9 vs 5–8), a higher number of subdigital lamellae on the first finger and on the first toe (FL1 4 or 5 vs 3; TL1 5 vs 3 or 4, respectively), fewer femoropre-cloacal pores in males (17 or 18 vs 20–26), the presence of dorsolateral pale-colored spots and dark dorsolateral stripe on trunk (vs absent), and a wider internarial width (SnW/HL 0.15–0.19 vs 0.13–0.14);

from *H. uga* Grismer, Wood, Thura, Zin, Quah, Murdoch, Grismer, Li, Kyaw & Lwin by having fewer circumnasal scales (CN 3 or 4 vs 5), more subdigital lamellae on the first finger and on the first toe (FL1 4 or 5 vs 2 or 3; TL1 5 vs 2 or 3, respectively), more lamellar formula on hand ((3 or 4)–4–4–4 vs (2 or 3)– (2 or 3)–3–3), more lamellar formula on foot (4–(4 or 5)–4–4 vs 3–3–3–3), fewer femoropre–cloacal pores in males (17 or 18 vs 18–22), and the presence of dorsolateral pale-colored spots, dark dorsolateral stripe on trunk, and postsacral marking bearing pale-colored anteriorly projecting arms (vs absent);

from *H. ywanganensis* Grismer, Wood, Thura, Zin, Quah, Murdoch, Grismer, Li, Kyaw & Lwin by having fewer chin scales (CS 8 or 9 vs 10), fewer circumnasal scales (CN 3 or 4 vs 5), more the number of subdigital lamellae on the first finger and on the first toe (FL1 4 or 5 vs 3; TL1 5 vs 2 or 3, respectively), more lamellar formula on hand ((3 or 4)–4–4–4 vs 3–3–(3 or 4)–3), more lamellar formula on foot (4–(4 or 5)–4–4 vs 3–3–3–3), fewer femoropre–cloacal pores in males (17 or 18 vs 26), and the presence of dark dorsolateral stripe on trunk (vs absent).

## Discussion

Phylogenetic analyses indicate that *Hemiphyllodactylus pakhaensis* sp. nov. is closely related to *H. simaoensis* and *H. menglianensis* (genetic pairwise distance 6.75% and 7.33%, respectively), which both occur in China. The type locality of the new species, Pa Kha 1 Village, Long Phieng Commune, Son La Province, northwestern Vietnam, lies ca 400 km from the type locality of *H. simaoensis* in Simao District, Pu’er City, Yunnan Province (Agung et al. 2022), and ca 520 km from that of *H. menglianensis* in Langdao Village, Menglian Dai, Lahu, and Wa Autonomous County, Yunnan Province. This considerable geographic separation supports the recognition of the new species as distinct from its closest relatives.

The discovery of this new species within Clade 4 represents the first record of this lineage from Vietnam, increasing the number of *Hemiphyllodactylus* species known from the country to 12. Nevertheless, the diversity of *Hemiphyllodactylus* in Vietnam is likely still underestimated, particularly in karst landscapes of northern Vietnam and in border areas between Vietnam and Laos. This is evidenced by the discovery of four new species from this region within the last two years: *H. vanhoensis* and *H. yenchauensis* from Vietnam, and *H. houaphanensis* and *H. xiengkhouangensis* from Laos (Luu et al. 2024, 2025). Therefore, further field research is required to uncover the unrealized diversity of *Hemiphyllodactylus* in the extensive karst formations of northern Vietnam and Laos.

The new species share conservation concerns as observed in *H. yenchauensis*, which was described from a nearby locality in Yen Chau District, Son La Province (Luu et al. 2025). Both *H. pakhaensis* sp. nov. and *H. yenchauensis* inhabit karst formations situated outside of existing protected areas. These regions are increasingly affected by agricultural expansion, deforestation, and human settlement, posing potential threats to their limited and highly localized populations. As both species appear to have narrow distribution ranges and are associated with disturbed karst habitats near human dwellings, their conservation status should be carefully assessed. Further ecological and population studies are urgently needed to evaluate their abundance, habitat specificity, and potential threats. Establishing long–term monitoring programs and considering the inclusion of their type localities into regional conservation planning would be essential steps to ensure the persistence of these narrowly endemic karst–dwelling geckos in northwestern Vietnam.

## Supplementary Material

XML Treatment for Hemiphyllodactylus pakhaensis

## Additional information

### Use of AI

No use of AI was reported.

### Author ORCIDs

Hong Bich Ha https://orcid.org/0000-0003-4777-5463

Tuoi Thi Hoang https://orcid.org/0000-0002-3564-0601

Manh Dac Nguyen https://orcid.org/0009-0002-6075-2274

Nghia Van Ha https://orcid.org/0000-0003-2515-8173

Vinh Quang Luu https://orcid.org/0000-0002-0634-1338

### Data availability

All of the data that support the findings of this study are available in the main text or Supplementary Information.

## Supplementary materials

Supplementary material 1 List of samples Data type xlsx Explanation note List of samples with GenBank accession numbers used for phylogenetic analyses in this study. All specimens are deposited in the Vietnam. https://binary.pensoft.net/file/1527382This dataset is made available under the Open Database License (http://opendatacommons.org/licenses/odbl/1.0/). The Open Database License (ODbL) is a license agreement intended to allow users to freely share, modify, and use this Dataset while maintaining this same freedom for others, provided that the original source and author(s) are credited. Hong Bich Ha, Tuoi Thi Hoang, Manh Dac Nguyen, Nghia Van Ha, Vinh Quang Luu

Supplementary material 2 Morphometric, meristic, and categorical data Data type xlsx Explanation note Morphometric, meristic, and categorical data used in the analyses for specimens of *Hemiphyllodactylus pakhaensis* sp. nov. and closely related *Hemiphyllodactylus* species (*H. simaoensis* and *H. menglianensis*); m = male; f = female. Bilateral meristic characters followed by ".5" represent averaged values. https://binary.pensoft.net/file/1527383This dataset is made available under the Open Database License (http://opendatacommons.org/licenses/odbl/1.0/). The Open Database License (ODbL) is a license agreement intended to allow users to freely share, modify, and use this Dataset while maintaining this same freedom for others, provided that the original source and author(s) are credited. Hong Bich Ha, Tuoi Thi Hoang, Manh Dac Nguyen, Nghia Van Ha, Vinh Quang Luu

Supplementary material 3 Summary statistics of morphometric and meristic characters Data type xlsx Explanation note Summary statistics of morphometric and meristic characters between *Hemiphyllodactylus pakhaensis* sp. nov. and closely related *Hemiphyllodactylus* species (*H. simaoensis* and *H. menglianensis*). https://binary.pensoft.net/file/1527384This dataset is made available under the Open Database License (http://opendatacommons.org/licenses/odbl/1.0/). The Open Database License (ODbL) is a license agreement intended to allow users to freely share, modify, and use this Dataset while maintaining this same freedom for others, provided that the original source and author(s) are credited. Hong Bich Ha, Tuoi Thi Hoang, Manh Dac Nguyen, Nghia Van Ha, Vinh Quang Luu

Supplementary material 4 Summary statistics and principal component analysis scores Data type xlsx Explanation note Summary statistics and principal component analysis scores between *Hemiphyllodactylus pakhaensis* sp. nov. and closely related *Hemiphyllodactylus* species (*H. simaoensis* and *H. menglianensis*). Dim1–16 of theMFA based on the total evidence dataset. Abbreviations are in the Materials and methods. https://binary.pensoft.net/file/1527385This dataset is made available under the Open Database License (http://opendatacommons.org/licenses/odbl/1.0/). The Open Database License (ODbL) is a license agreement intended to allow users to freely share, modify, and use this Dataset while maintaining this same freedom for others, provided that the original source and author(s) are credited. Hong Bich Ha, Tuoi Thi Hoang, Manh Dac Nguyen, Nghia Van Ha, Vinh Quang Luu
